# Meaning and Purpose (MaP) therapy I: Therapeutic processes and themes in advanced cancer – CORRIGENDUM

**DOI:** 10.1017/S1478951526102715

**Published:** 2026-06-10

**Authors:** Carrie Lethborg, David W. Kissane, Penelope Schofield

**Keywords:** Advanced cancer, Distress, Meaning-based intervention, Sense of significance, Suffering, Therapeutic processes, Unique meaning, corrigendum

The authors apologise for the following oversight.

In: Lethborg, C., Kissane, D.W., & Schofield, P. (2019), *‘Meaning and Purpose (MaP) therapy I: Therapeutic processes and themes in advanced cancer’*, a quotation attributed to “Participant 1” (p.5) was inadvertently reproduced from the 2012 article listed below (*Lethborg, Schofield, & Kissane, 2012*), where it was attributed to a participant from the developmental phase of the intervention.

This quotation does not originate from the pilot cohort (n=24) described in the 2019 study and was included in error.

The qualitative analysis and findings reported in the 2019 article are based solely on data from the pilot study cohort and are not affected by this error. In qualitative research, such quotations are used to illustrate themes derived from the analytic dataset rather than to constitute the analysis itself; accordingly, this error does not affect the reported findings.

The articles in question are:

1. Lethborg, C., Schofield, P., & Kissane, D. (2012). The advanced cancer patient experience of undertaking meaning and purpose (MaP) therapy. *Palliative and Supportive Care*, 10, 177-188. doi: 10.1017/S147895151100085X
2. Lethborg C, Kissane DW, Schofield P (2019). Meaning and Purpose (MaP) therapy I: Therapeutic processes and themes in advanced cancer. *Palliative and Supportive Care*, 17, 13-20. doi: 10.1017/S1478951518000871
